# Metabolic syndrome and insulin resistance in migraine

**DOI:** 10.1007/s10194-012-0416-y

**Published:** 2012-01-26

**Authors:** Sanjeev K. Bhoi, Jayantee Kalita, Usha K. Misra

**Affiliations:** Department of Neurology, Sanjay Gandhi Post Graduate Institute of Medical Sciences, Raebareily Road, Lucknow, 226014 India

**Keywords:** Migraine, Obesity, Metabolic syndrome, Insulin resistance, HOMA

## Abstract

Metabolic syndrome is associated with migraine but there is no study comparing the characteristics of migraine with and without metabolic syndrome from Southeast Asia. This study was therefore undertaken to compare the clinical characteristics of migraine in patients with and without metabolic syndrome and insulin resistance. 135 consecutive patients with migraine diagnosed on the basis of International Headache Society criteria were subjected to clinical evaluation as per fixed protocol. Headache severity, frequency and functional disability were recorded. Metabolic syndrome was diagnosed as per National Cholesterol Education Programme: Adult Treatment Panel III and International Diabetic Federation criteria. Insulin resistance was calculated by homeostases model assessment. Their age ranged between 14 and 61 years and 108 were females. Metabolic syndrome was present in 31.9% patients and only 13 were obese. Insulin resistance was present in 11.1%. Metabolic syndrome was correlated with age, gender, number of triggers, years of headache and duration of migraine attacks. Insulin resistance correlated with duration of migraine attacks. From this study, it can be concluded that metabolic syndrome was present in 31.9% of the migraineurs which was mainly in elderly who had longer duration of headache and multiple triggers.

## Introduction

Migraine is a common disorder with overall prevalence of 5–15% [1–3].The prevalence of migraine in females is higher (6–22%) compared to males (3–7%) [4–6]. The pathogenesis of migraine is multifactorial and a number of genetic and environmental factors have been suggested. Recently, association of migraine with metabolic syndrome and insulin resistance has been reported [7, 8]. The prevalence of migraine was estimated as 11.9% in males and 22.5% in females with metabolic syndrome. Of the components of metabolic syndrome diabetes mellitus, increased waist circumference and body mass index (BMI) were more frequent in migraine patients compared to those without migraine. The frequency of hypertension and dyslipidemia, however, was not significantly different [9]. In a population-based study on obesity and migraine evaluating 30,215 participants whose mean age was 38.4 years including 65% females, revealed lack of association of BMI with the prevalence of migraine but was associated with the frequency of headache attacks. In the normal weight group, 4.4% had 10–15 headache days per month increasing to 5.8% in the overweight and 20.7% in the morbidly obese [10]. Majority of these studies are from the western countries where obesity is common. In a population-based study, 685 women aged 40–74 years were evaluated for relationship of BMI and migraine. The proportion of obesity (BMI >30) did not differ in the women with active migraine, inactive migraine and those who never experienced migraine. The frequency, duration and severity of migraine attacks also did not differ between obese and non-obese migraine patients [11].

On PubMed search using Keywords “Migraine” and “Metabolic syndrome/insulin resistance/insulin metabolism” revealed six articles; two on metabolic syndrome and four on insulin metabolism in migraine [8, 9, 12–15]. The incidence of obesity is lower in Asian countries and we could not find any study from Southeast Asia evaluating the role of metabolic syndrome in migraine. In the present study, we report the frequency of metabolic syndrome in migraine patients and compare the clinical characteristics of migraine in patients with and without metabolic syndrome as well as insulin resistance.

## Patients and methods

The patients with migraine were recruited from the neurology out patient service of Sanjay Gandhi Postgraduate Institute, Lucknow, India during January 2009–June 2010. The diagnosis of migraine was based on International Headache Society Criteria [16]. The children below 13 years of age were excluded because ours is an adult neurology service. All the patients were subjected to medical history and clinical evaluation as per a fixed protocol. Family history of headache in three generations was noted. Migraine triggers were enquired using a questionnaire evaluating various exogenous (sun exposure, noise, cold, hot, weather change, travelling, hair wash, dry hair, applying hair oil and food items) and endogenous triggers (physical and mental stress, fasting for 12 h or more, hunger, sleep deprivation and menstruation). The presence of mechanical (static and dynamic) and thermal allodynia was recorded from checklist which included combing hair, fan air, shower, shaving, resting head on pillow, wearing neck tie, ornaments, hair clip, scurf, veil and ear ring, hot and cold during the interview. The extent of allodynia was categorized into cephalic only and extension to extracephalic region (neck and upper limb). The duration of migraine in years, its frequency per month, associated symptoms, functional disability, duration of each attack in days and response to treatment were evaluated. The severity of headache was graded on a 0–3 scale (0 = normal, 1 = mild, 2 = moderate, 3 = severe headache). We considered average severity of headache in the last month. The functional disability was graded on 0–4 scale [0 = normal, 1 = mild impairment of activities of daily living (ADL), 2 = moderate, 3 = severe and 4 = inability to perform ADL activities or bedridden state]. Presence of aura and associated symptoms such as nausea, vomiting, photophobia and phonophobia were also evaluated. The migraine index was calculated by average number of attacks per month multiplied by average severity of headache. Corrected migraine index was calculated by migraine index multiplied by average duration of attack.

Diagnosis of metabolic syndrome was based on National Cholesterol Education Programme (NCEP): Adult Treatment Panel (ATP) III 2001 [17] and International Diabetic Federation (IDF) criteria [18], according to which three of the following criteria were needed including central obesity.

1. Central obesity: waist circumference >90 cm in males and 80 cm in females.
2. Hypertriglyceridemia >150 mg/dl or on specific medication.
3. Low HDL cholesterol <40 mg/dl in males and <50 mg/dl in females.
4. Hypertension: blood pressure >130 systolic or >85 mm Hg diastolic or specific medication.
5. Fasting plasma glucose >100 mg/dl or on specific medication or previously diagnosed type 2 diabetes.

Insulin resistance and β cell function were calculated in 90 patients by HOMA model [19].

$$ {\text{HOMA-IR}}\,\left( {\text{insulin resistance}} \right) \, = \, \frac{{{\text{Fasting\, blood\, sugar }} \times {\text{ fasting\,insulin}}}}{ 40 5} $$

$$ {\text{HOMA-B}}\,\left( {\beta {\text{ cell function}}} \right){\text{ was calculated as follows}}:\, = \frac{{ 3 60 \, \times {\text{ insulin}}}}{\text{Fasting blood sugar}} \, - { 63}$$

The cut-off value of insulin resistance was taken as >1.77 for the diagnosis of metabolic syndrome [20].

Blood counts, haemoglobin, ESR, platelet count, blood urea nitrogen, serum creatinine, bilirubin, transaminases and TSH were carried out. Fasting and 2-h post prandial blood sugar, lipid profile and insulin level were estimated. The blood sugar was assayed by autoanalyzer using oxidase–peroxidase method. The insulin was estimated by immunoradiometric assay using Siemens Medical Solutions Diagnostic, LA, USA. Patients with high thyroid stimulating hormone, renal and hepatic failure were excluded.

### Statistical analysis

The migraine patients were categorized into those with metabolic syndrome and those without. The patients were also categorized into insulin resistant and those without based on HOMA-IR. The demographic, severity and frequency of migraine, presence of allodynia, aura and triggers were compared in those with and without metabolic syndrome as well as with and without insulin resistance using *X* ^2^ and independent *t*’ test. The variables were considered significant if 2-tailed *p* value was <0.05. The statistical analysis was done using SPSS 15 version software.

## Results

There were 135 patients with migraine whose mean age was 31.4 (SD 10.5) years; 108 of whom were females. Family history of migraine was present in 58 patients. The mean duration of headache was 8.5 (SD 6.7) years. Only seven patients had migraine with aura. The frequency of migraine ranged between 2 and 30 attacks per month; 54 of them had headache frequency of more than 15 attacks per month. Mechanical (static and dynamic) allodynia was present in 116 (85.9%) and 27 (20%) of them also had thermal allodynia. The allodynia was cephalic in 43 (37.1%) and extended to extracephalic region in 73 (62.9%) patients. The majority of patients [134 (99.3%)] had moderate to severe headache. The general characteristics of the patients are provided in Table 1.

**Table 1:** Demography and clinical characteristics of migraine patients

Parameters	Number of patients (*n* = 135)
Age (years)	31.41 ± 10.5 (range 14–61)
Female	108 (80%)
Urban/rural	81/54
Education (years)	10.36 ± 5.02
Family history of headache	58 (43%)
Total family members affected	1.66 ± 0.9 (range 1–6)
Only patients	77 (57%)
1st generation	33 (24.4%)
2nd generation	23 (17%)
3rd generation	2 (1.5%)
Migraine with aura	7 (5.2%)
Duration (years)	8.48 ± 6.7 (range 0.5–40)
Frequency/months	15.4 ± 10.7 (range 2–30)
Duration attack (days)	1.8 ± 0.8 (range 0.5–4)
Total no. of triggers	10.84 ± 2.78 (range 2–15)
Allodynia	116 (85.9%)
Mechanical	116 (85.9%)
Static	116 (85.9%)
Dynamic	115 (85.2%)
Thermal	27 (20%)
Cephalic	43 (37.1%)
Extracephalic	73 (62.9%)
Number of metabolic parameters abnormality (0–5)	
0	11 (8.1%)
1	41 (30.4%)
2	40 (29.6%)
3	26 (19.3%)
4	14 (10.4%)
5	3 (2.2%)

### Metabolic syndrome

The BMI was above 30 in 13 (9.6%) patients and between 25 and 30 in 26 (19.3%). Female patients were more overweight compared to males (34 vs. 5) and 2 females were morbidly obese (BMI >35). Waist circumference was abnormal in 78 (57.8%) patients. HDL was reduced in 108 (80%) and triglyceride was increased in 28 (20.7%) patients. Fasting blood sugar was high in 13 (9.6%) patients. The systolic blood pressure was raised in 15 and diastolic in 33 (24.5%) patients. Insulin resistance (HOMA-IR) was present in 10 (11.1%) patients. β cell function (HOMA-B) was 179.3 ± 552.4. Based on ATP-III and IDF criteria, 43 (31.9%) patients had metabolic syndrome. Out of 5 components of metabolic syndromes, all 5 were present in 3, 4 in 14 and 3 in 26 patients. Only 2 components of metabolic syndrome were present in 40 patients, however they were not diagnosed as metabolic syndrome.

### Correlation

The patients with metabolic syndrome were older (*p* = 0.0001), more frequently females (*p* = 0.01), had longer duration of illness (10.3 ± 7.5 vs. 7.6 ± 6.2, *p* = 0.03), higher number of triggers (11.6 ± 2.5 vs. 10.5 ± 2.8, *p* = 0.03) and longer duration of attack (2.0 ± 0.8 vs. 1.7 ± 0.8, *p* = 0.05) compared to those without metabolic syndrome. The majority of patients had multiple triggers; commonest being noise in 129 (95.6%) followed by stress in 128 (94.8%), sunlight in 121 (89.6%) and fasting in 100 (74.1%) patients. On comparing the triggers, head wash was more frequent in those with metabolic syndrome compared to without (65.1% vs. 44.6%; *p* = 0.03). The details of triggers are given in Table 2. The presence of aura (3 vs. 4, *p* = 0.68), frequency of headache (15.1 ± 11.1 vs. 15.5 ± 10.6, *p* = 0.82), severity of migraine (*p* = 0.66), migraine index (MI) (30.8 ± 22.6 vs. 37.0 ± 28.0, *p* = 0.20), corrected migraine index (MIc) (55.8 ± 39.4 vs. 54.6 ± 40.0, *p* = 0.87), functional impairment (*p* = 0.19), presence of allodynia (*p* = 0.79) and extent of allodynia (*p* = 0.84) were not significantly different between the two groups (Table 3). Insulin resistance (HOMA-IR >1.77) was present in 10 (11.1%) patients. There was no difference in demography, duration of migraine, migraine index, corrected migraine index, number of triggers, and fasting and hunger as triggers between the two groups (Table 4). The duration of migraine attack was more in the patients with insulin resistance (*p* = 0.04).

**Table 2:** Frequency of different triggers migraine and its comparison in the patients with and without metabolic syndrome

Triggers	Total number of patients (%)	Metabolic syndrome	Metabolic syndrome	*p* value
		Present	Absent	
		*N* = 43 (31.9%)	*N* = 92 (68.1%)	
Physical stress	128 (94.8)	41	87	1.00
Mental stress	128 (94.8)	40	88	0.70
Fasting	100 (74.1)	36	64	0.09
Hunger	100 (74.1)	36	64	0.09
Sunlight	121 (89.6)	41	80	0.22
Noise	129 (95.6)	43	86	0.18
Cold exposure	81 (60)	23	58	0.35
Hot exposure	100 (74.1)	34	66	0.41
Weather change	104 (77)	35	69	0.51
Menstruation	35 (30.7)	12	23	1.00
Sleep deprivation	119 (88.1)	40	79	0.27
Food	6 (4.4)	3	3	0.38
Travel	116 (85.9)	40	76	0.12
Perfume	33 (24.4)	11	22	0.83
Hair oil	3 (2.2)	1	2	1.00
Head wash	69 (51.1)	28	41	0.03
Dry hair	88 (65.2)	33	55	0.08

**Table 3:** Comparison of clinical characteristics in patients with and without metabolic syndrome

Parameters	Metabolic syndrome	Metabolic syndrome	*p* value
	Present	Absent	
	*N* = 43 (31.9%)	*N* = 92 (68.1%)	
Age (years)	37.8 ± 7.7	28.4 ± 10.3	<0.0001
Female	40 (93%)	68 (73.9%)	0.01
Urban/rural	26/17	55/37	1.00
Education (years)	9.35 ± 4.76	10.84 ± 5.09	0.11
Family history of headache	22 (51.2%)	36 (39.1)	0.20
Total family members affected	1.86 ± 1.15	1.57 ± 0.79	0.08
Migraine with aura	3 (7%)	4 (4.4%)	0.68
Duration (years)	10.28 ± 7.5	7.64 ± 6.21	0.03
Total no. of triggers	11.58 ± 2.53	10.49 ± 2.84	0.03
Fasting	36 (83.7%)	64 (69.6%)	0.09
Hunger	36 (83.7%)	64 (69.6%)	0.09
Onset to peak time (min)	32.09 ± 32.68	27.61 ± 20.17	0.33
Frequency/months	15.07 ± 11.05	15.52 ± 10.58	0.82
Duration attack (days)	2.0 ± 0.75	1.72 ± 0.78	0.05
Severity of headache	2.98 ± 0.15	2.93 ± 0.29	0.37
Functional impairment	3.28 ± 0.7	3.2 ± 0.62	0.48
Rescue analgesics/mo	13.07 ± 9.82	14.03 ± 11.58	0.64
Allodynia	38 (88.4%)	78 (84.8%)	0.79
Migraine index (MI)	30.75 ± 22.59	37.03 ± 27.97	0.20
Corrected MI (MIc)	55.83 ± 39.43	54.6 ± 40.04	0.87

**Table 4:** Comparison of clinical characteristics in patients with and without insulin resistance (HOMA-IR >1.775)

Parameters	Insulin resistance	Insulin resistance	*p* value
	Present	Absent	
	*N* = 10 (11.1%)	*N* = 80 (88.9%)	
Age (years)	33.0 ± 9.04	31.39 ± 10.98	0.66
Female	8 (80%)	62 (77.5%)	1.00
Urban/rural	6/4	45/35	0.81
Education (years)	11.0 ± 3.86	10.05 ± 5.06	0.57
Migraine with aura	0(%)	6 (7.5%)	0.37
Duration (years)	9.98 ± 6.30	8.40 ± 7.48	0.52
Total no. of triggers	10.9 ± 3.45	11.15 ± 2.54	0.78
Fasting	7 (70%)	59 (73.8%)	0.72
Hunger	7 (70%)	59 (73.8%)	0.72
Onset to peak	28.0 ± 13.78	27.00 ± 17.73	0.86
Severity of headache	3.00 ± 00	2.95 ± 0.27	0.56
Severity of functional impairment	3.10 ± 0.88	3.16 ± 0.63	0.78
Frequency/months	16.00 ± 12.27	13.53 ± 10.7	0.50
Duration attack (days)	2.42 ± 0.66	1.90 ± 0.78	0.04
Rescue analgesics/months	17.10 ± 14.43	12.39 ± 9.77	0.18
Allodynia	9 (90%)	69 (86.3%)	1.00
Family history of headache	6 (60%)	31 (38.6%)	0.31
Migraine index (MI)	35.40 ± 25.97	31.46 ± 25.98	0.65
Corrected MI (MIc)	75.26 ± 42.07	52.65 ± 43.01	0.12
No. of metabolic parameters abnormality (metabolic syndrome)	3.70 ± 1.16	1.48 ± 0.98	<0.0001

## Discussion

In the present study, 31.9% of the migraine patients had metabolic syndrome but only 9.6% were obese. The diagnosis of metabolic syndrome was based on increased waist circumference (57.8%), hypertriglyceridemia (20.7%), hypertension (27.4%), low HDL (80.0%) and increased fasting blood sugar (9.6%). Moreover, insulin resistance was present in 11.1% patients. These observations are in agreement with the reports from the western countries in which metabolic syndrome has been associated with migraine [7, 9, 21]. In our study, only 2 patients were morbidly obese and remaining 11 patients were obese. In a study from USA, the effect of BMI on the prevalence of severe headache or migraine revealed significant association of headache or migraine with low (<18.5 kg/m^2^) and high BMI (>30 kg/m^2^) [22]. In Asians, central obesity is common and was present in 57.8% of our patients. Central obesity, high triglyceride and low HDL are reported to be the main component of metabolic syndrome in the Asians [23], whereas in the western countries high BMI is more common [13, 24].

Metabolic syndrome is associated with insulin resistance. The patients with migraine have significantly higher level of fasting glucose and insulin; both of which remain elevated after glucose loading suggesting insulin resistance [14]. Similar observations have also been reported in a smaller study in which oral glucose tolerance test and plasma glucose were significantly higher in migraine patients compared to controls. Insulin sensitivity is significantly altered in migraine patients as measured by ISI-Stumvoll and OGIS-180 index [8]. Insulin resistance is a state in which normal amount of insulin produces a subnormal physiological response. Insulin resistant state may increase free fatty acids and blood lipids which may induce migraine attack [25]. Low HDL is an important component of metabolic syndrome and HDL has anti-inflammatory property. Reduced LDL may reduce pain perception [26].

In our study, metabolic syndrome was significantly related to age. The mean age in the patients with metabolic syndrome was 37.8 (SD 7.7) years, whereas it was 28.4 (SD 10.3) years in those without. Age may be directly related to various components of metabolic syndrome such as obesity, hypertension, glucose intolerance and hyperlipidemia which may be an association rather than underlying mechanism. In our study, metabolic syndrome and migraine both were more common in females than males. Whether it is merely an association or it has a biological basis needs further study. Migraine is precipitated by a number of endogenous and exogenous triggers. Fasting and hunger are important triggers and have been reported in Indian subjects in 46.3% [27]. Fasting is common in both Hindus and Muslims as a religious practice. In the present study, the most common trigger was noise (95.6%) followed by stress (94.8%), sun exposure (89.6%) and fasting (74.1%). Fasting results in activation of insulin receptors which may trigger a migraine attack [28]. Low sucrose diets may reduce the frequency of migraine attack [29]. In migraineurs, the onset of diabetes mellitus increases the average yearly number of headache days [30]. In our study, fasting was neither associated with metabolic syndrome nor with insulin resistance but the duration of migraine attack (days) was significantly related to insulin resistance. Our results are limited by absence of a control group, absence of children, disproportionately large number of females and unequal number of patients with and without metabolic syndrome. In a population-based study from North India, the prevalence of metabolic syndrome is 67.2% (male 75.9% and female 58.4%) and obesity in 79.3% (male 74.5% and female 84.0%) [31]. The lower frequency of metabolic syndrome and obesity in our study compared to the above-mentioned study may be due to the referral bias. The study by Yadav et al. [31] was based on urban affluent population. We have studied migraine patients from different regions of North India including both urban and rural population comprising diverse socioeconomic classes.

Based on our results, it can be concluded that migraine is associated with metabolic syndrome in 31.9% and insulin resistance in 11.1% patients. The patients with metabolic syndrome are older, have multiple triggers, longer history of migraine as well as duration of migraine attack. A larger controlled study is needed to confirm these results.
